# Antibody-Based Inhibition of Pathogenic New World Hemorrhagic Fever Mammarenaviruses by Steric Occlusion of the Human Transferrin Receptor 1 Apical Domain

**DOI:** 10.1128/JVI.01868-20

**Published:** 2021-08-10

**Authors:** Sol Ferrero, Maria D. Flores, Connor Short, Cecilia A. Vazquez, Lars E. Clark, James Ziegenbein, Samantha Zink, Daniel Fuentes, Cristian Payes, María V. Batto, Michael Collazo, Cybele C. García, Jonathan Abraham, Sandra M. Cordo, Jose A. Rodriguez, Gustavo Helguera

**Affiliations:** a Laboratory of Pharmaceutical Biotechnology, Instituto de Biología y Medicina Experimental (IBYME-CONICET), Buenos Aires, Argentina; b Department of Chemistry and Biochemistry, UCLA-DOE Institute for Genomics and Proteomics, University of California Los Angeles, Los Angeles, California, USA; c Laboratorio de Virología, Departamento de Química Biológica, Facultad de Ciencias Exactas y Naturales, Universidad de Buenos Aires (UBA), IQUIBICEN, Consejo Nacional de Investigaciones Científicas y Técnicas (CONICET)-UBA, Ciudad Universitaria, Buenos Aires, Argentina; d Department of Microbiology, Blavatnik Institute, Harvard Medical School, Boston, Massachusetts, USA; e Department of Biological Chemistry and Department of Chemistry and Biochemistry, University of California Los Angeles, Howard Hughes Medical Institute, UCLA-DOE Institute for Genomics and Proteomics, Los Angeles, California, USA; f Department of Medicine, Division of Infectious Diseases, Brigham and Women’s Hospital, Boston, Massachusetts, USA; g IQUIBICEN, Consejo Nacional de Investigaciones Científicas y Técnicas (CONICET)-UBA, Ciudad Universitaria, Buenos Aires, Argentina; University of Kentucky College of Medicine

**Keywords:** Sabiá-like, X-ray crystallography, antiviral agents, electron microscopy, Junin, Machupo, mammarenavirus, monoclonal antibodies, transferrin receptor

## Abstract

Pathogenic clade B New World mammarenaviruses (NWM) can cause Argentine, Venezuelan, Brazilian, and Bolivian hemorrhagic fevers. Sequence variability among NWM glycoproteins (GP) poses a challenge to the development of broadly neutralizing therapeutics against the entire clade of viruses. However, blockade of their shared binding site on the apical domain of human transferrin receptor 1 (hTfR1/CD71) presents an opportunity for the development of effective and broadly neutralizing therapeutics. Here, we demonstrate that the murine monoclonal antibody OKT9, which targets the apical domain of hTfR1, can sterically block cellular entry by viral particles presenting clade B NWM glycoproteins (GP1-GP2). OKT9 blockade is also effective against viral particles pseudotyped with glycoproteins of a recently identified pathogenic Sabia-like virus. With nanomolar affinity for hTfR1, the OKT9 antigen binding fragment (OKT9-Fab) sterically blocks clade B NWM-GP1s and reduces infectivity of an attenuated strain of Junin virus. Binding of OKT9 to the hTfR1 ectodomain in its soluble, dimeric state produces stable assemblies that are observable by negative-stain electron microscopy. A model of the OKT9-sTfR1 complex, informed by the known crystallographic structure of sTfR1 and a newly determined structure of the OKT9 antigen binding fragment (Fab), suggests that OKT9 and the Machupo virus GP1 share a binding site on the hTfR1 apical domain. The structural basis for this interaction presents a framework for the design and development of high-affinity, broadly acting agents targeting clade B NWMs.

**IMPORTANCE** Pathogenic clade B NWMs cause grave infectious diseases, the South American hemorrhagic fevers. Their etiological agents are Junin (JUNV), Guanarito (GTOV), Sabiá (SABV), Machupo (MACV), Chapare (CHAV), and a new Sabiá-like (SABV-L) virus recently identified in Brazil. These are priority A pathogens due to their high infectivity and mortality, their potential for person-to-person transmission, and the limited availability of effective therapeutics and vaccines to curb their effects. While low homology between surface glycoproteins of NWMs foils efforts to develop broadly neutralizing therapies targeting NWMs, this work provides structural evidence that OKT9, a monoclonal antibody targeting a single NWM glycoprotein binding site on hTfR1, can efficiently prevent their entry into cells.

## INTRODUCTION

Despite the gravity of South American hemorrhagic fevers (1–3), treatments against pathogenic New World mammarenaviruses (NWMs) remain limited. Low sequence homology among the glycoproteins of this family of viruses hinders the development of broadly neutralizing virus-targeting therapeutics. A vaccine against Argentine hemorrhagic fever (AHF) derived from attenuated JUNV strain CANDID#1 is produced in Argentina and has reduced incidence of the disease, but to date no effective FDA-approved treatment or vaccine exists against all New World hemorrhagic fevers (NWHFs) (4). Small molecules have shown limited efficacy against NWHF viruses; the antiviral favipiravir (T-705) in combination with rivabirin (5) and RNA aptamers are under investigation but have yet to reach clinical trials (44). Recent efforts have found success in the development of neutralizing monoclonal antibodies against NWHF mammarenavirus glycoproteins. However, while virus-neutralizing monoclonal antibodies can mimic important receptor contacts when engaging the glycoprotein of a particular NWM (6), they show limited or no activity against the glycoproteins of other members of the NWM family (4, 6).

Despite the low sequence identity between their glycoprotein sequences (25% to 46%), clade B NWMs share routes of entry into human cells. One such example is the human transferrin receptor (hTfR1), a single-pass transmembrane protein that regulates iron uptake into cells (7). Structural studies have determined the atomic nature of the interaction of Machupo virus (MACV) GP1 with hTfR1 and revealed a conserved binding site for clade B NWM GP1 binding on the apical domain of hTfR1 (8). The putative binding of all members of this clade to the same epitope on hTfR1 makes it an attractive molecular target for the development of broad-spectrum inhibitors of viral entry, including antibodies (9). Recombinant monoclonal antibodies have been identified that exploit this vulnerability by binding hTfR1 and blocking the internalization of pseudotyped viral particles decorated with Junin (JUNV), Guanarito (GTOV), Sabiá (SABV), MACV, Chapare (CHAV), and Sabiá (SABV) virus glycoproteins (GP) but not pseudotyped viral particles expressing glycoproteins from Old World hemorrhagic fever viruses, such as Lassa virus (LASV) (9). More recently, such antibodies have been shown to block entry of the North American mammarenavirus AV96010151 into cells, expanding their potential efficacy against a broader spectrum of NWHF viruses (45). Likewise, arenacept, a recombinant protein consisting of the apical domain of hTfR1 fused to an Fc domain, is capable of binding GP1 of several NWMs and preventing pseudotyped virus internalization into cells (10).

While hTfR1-targeting antibodies have demonstrated potential as broadly neutralizing therapeutics against clade B NWMs, this strategy faces key challenges. A concrete molecular basis for the inhibition of viral entry through hTfR1-targeting antibodies is still lacking. This is due in part to the absence of structures of these targeting agents bound to hTfR1 despite a large number being discovered and explored for other therapeutic applications. The murine monoclonal OKT9 was among the first antibodies shown to recognize hTfR1 in several cell lines and have its activity explored in a variety of contexts (11–13). We now demonstrate that OKT9 is a potential blocker of cell entry by clade B NWMs, as it engages an epitope on the apical domain of hTfR1 that is a shared binding site for clade B NWM glycoproteins. OKT9 prevents cell entry by pseudotyped viral particles with the surface glycoproteins of a newly sequenced and lethal Sabiá-like (SABV-L) NWM (14). We also demonstrate that OKT9 blockade can inhibit the *in vitro* replication of a competent, albeit attenuated, strain of JUNV (15). Knowledge that OKT9 blocks the GP1-TfR1 interaction through steric occlusion presents a mechanistic platform for the development of broadly active competitive inhibitors of NWHF that target viral entry mediated by hTfR1.

## RESULTS

### The OKT9 variable region binds hTfR1 with nanomolar affinity.

**FIG 1:**
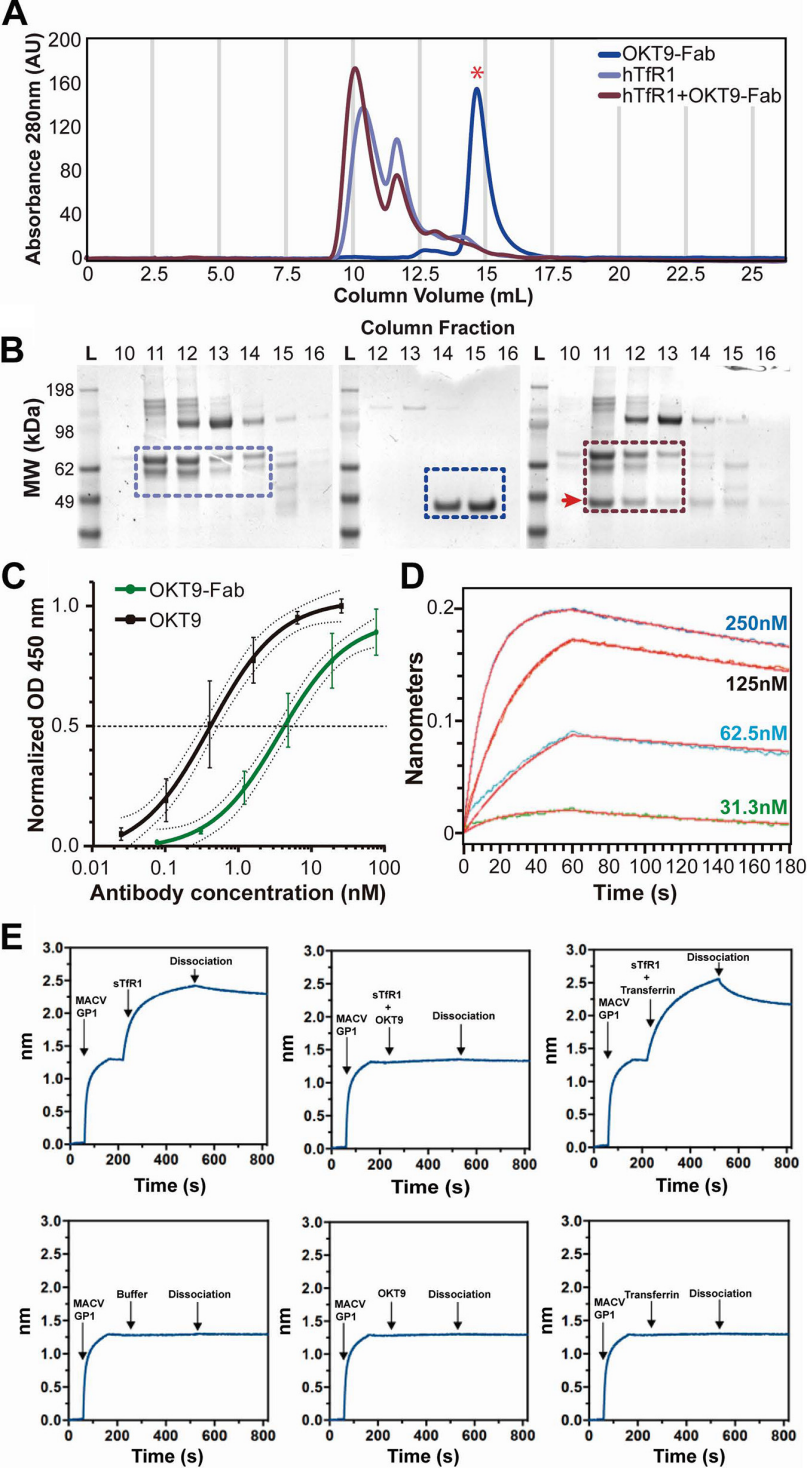
Characterization of OKT9 binding to hTfR1. (A) Formation of an hTfR1-OKT9 complex evaluated by SEC coelution. Samples include hTfR1 (purple), OKT9-Fab (blue), and sTfR1+OKT9-Fab (maroon). An overlay of all traces is shown for comparison. Gray bars indicate collected fractions. The red star denotes the expected peak fraction for OKT9-Fab alone. (B) SDS-PAGE of fractions collected from isolated and complex SEC runs. On the left is shown the SDS-PAGE of the SEC fractions of the hTfR1 run, in the center the fractions of the OKT9-Fab run, and on the right the fractions of the complex. Colored boxes indicate the presence of the protein of interest, coordinated with the color used in SEC traces. The red arrow denotes the expected band for OKT9-Fab. (C) ELISA binding of OKT9 to hTfR1. An indirect ELISA was performed decorating the plate with sTfR1 and then incubating with different concentrations of OKT9-Fab and OKT9. Anti-mouse IgG conjugated to HRP was used as a secondary antibody. The EC_50_ calculated over the normalized OD_450_ for OKT9 was 0.411 nM with a 95% confidence interval (CI) of 0.213 to 0.633, and for OKT9-Fab the EC_50_ was 3.695 nM with a 95% CI of 2.463 to 6.028. (D) Kinetics of OKT9-Fab interaction with hTfR1 immobilized on a biosensor surface. The receptor was exposed to increasing concentrations of OKT9-Fab as labeled (31.3 nM, 62.5 nM, 125 nM, and 250 nM). One hundred eighty seconds of biolayer recordings show binding and dissociation. (E) Assessment of OKT9-Fab and MACV GP1-Fc for binding to sTfR1 by biolayer interferometry. MACV GP1-Fc immobilized onto anti-human Fc biosensor tips and incubated with sTfR1 (top left) or buffer alone (bottom left), sTfR1 in complex with OKT9-Fab (top center), OKT9-Fab alone (bottom center), sTR1 in complex with transferrin (top right), or transferrin alone (bottom right). The arrows indicate the time points at which the indicated proteins were added and the dissociation step. The data are representative of two replicates for each of the experimental conditions shown.

Despite the longstanding characterization of OKT9 as a monoclonal antibody that binds hhTfR1, we first set out to biochemically assess this interaction using a recombinantly generated soluble form of hTfR1 with a C-terminal hexahistidine tag that we refer to as hTfR1 (16). Size-exclusion chromatography (SEC) of hTfR1 yields peaks that elute at column fractions consistent with monomer and multimer species in solution; sampling of peak fractions and evaluation by SDS-PAGE shows a single principal band at ∼79 kDa, corresponding to a monomer. Traces for OKT9-Fab alone show a principal peak eluting at column fractions consistent with a monomer; a 50-kDa band is observed from these fractions by SDS-PAGE, consistent with the expected molecular weight for a Fab monomer (Fig. 1A). hTfR1 was mixed with OKT9-Fab at a 3:1 molar ratio and incubated for 20 min at room temperature to facilitate the formation of a solution-state complex. Traces of the mixture show a shift of peaks to earlier column fractions compared to the receptor alone. The peak corresponding to OKT9-Fab alone disappears in traces for the mixture, indicating a loss of free Fab in solution. This is confirmed by SDS-PAGE of peak fractions showing a coelution of Fab with TfR1 in early fractions (Fig. 1B).

We evaluated the binding of full-length OKT9 and its proteolytic antigen binding fragment (OKT9-Fab) to hTfR1 immobilized on a solid surface or in solution. Both OKT9 and OKT9-Fab bound surface-immobilized hTfR1 in a concentration-dependent manner with approximate 50% effective concentration (EC_50_) values of 0.411 nM (OKT9) and 3.659 nM (OKT9-Fab) (Fig. 1C). The affinity of OKT9-Fab for hTfR1 was further assessed by biolayer interferometry (BLI), with various concentrations of full-length OKT9 exposed to a fixed concentration of hTfR1 immobilized on the biolayer sensor via an anti-His antibody and assuming a 1:1 interaction between Fab and receptor. Averaging across four biolayer cycles produced an equilibrium dissociation constant of 4.8 nM for the interaction between OKT9-Fab and hTfR1 (Fig. 1D). Rate constants for each cycle are shown in Table 1. Biolayer interferometry was also used to directly evaluate competition between OKT9-Fab and a MACV GP1-Fc fusion protein for binding to a soluble form of hTfR1 (sTfR1) in solution (Fig. 1E). MACV GP1-Fc bound to a biosensor surface was able to efficiently bind hTfR1, but this binding was abrogated in the presence of OKT9-Fab. By comparison, transferrin, which does not compete with MACV for binding to hTfR1 (7), had no effect on hTfR1 binding to immobilized MACV GP1-Fc. These results suggest that OKT9 and MACV GP1 bind overlapping sites on hTfR1.

**TABLE 1:** Kinetics analysis of OKT9-Fab binding to hTfR1 via bilayer interferometry^*a*^

Cycle	*K*_D_ (M)	*K*_D_ error	*k_a_* (1/ms)	*k_a_* error	*K*_dis_ (1/s)	*K*_dis_ error	Full *X*^2^	Full *R*^2^
A	4.76e−09	8.32e−11	2.38e+05	1.68e+03	1.14e−03	1.81e−05	0.0728	0.9951
B	4.82e−09	8.00e−11	2.50e+05	1.73e+03	1.20e−03	1.82e−05	0.0730	0.9954
C	4.90e−09	8.15e−11	2.54e+05	1.81e+03	1.25e−03	1.87e−05	0.0767	0.9952
D	4.78e−09	7.59e−11	2.62e+05	1.78e+03	1.25e−03	1.80e−05	0.0695	0.9956

a *K*_D_, equilibrium dissociation constant; *k_a_*, association rate constant; *K*_dis_, dissociation rate constant.

### OKT9 and OKT9-Fab block cellular entry of pseudotyped viruses presenting clade B NWM glycoproteins and a nonpathogenic strain of JUNV.

**FIG 2:**
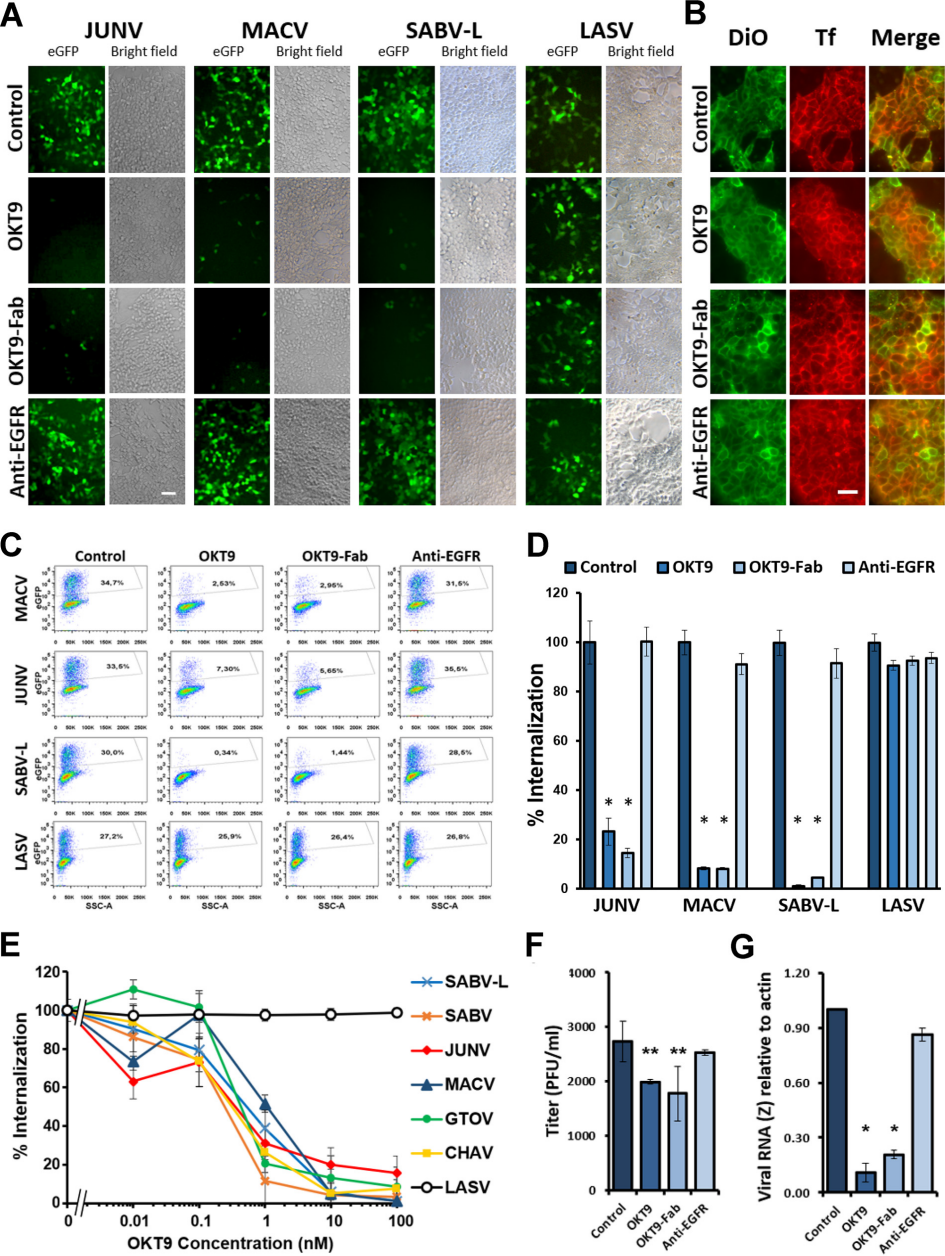
Inhibition of internalization of NWHF pseudovirus by OKT9. (A) Fluorescence microscopy images of HEK-293T cells. The images were taken from the inhibition of JUNV, MACV, SABV-L, and LASV pseudovirus internalization assays mentioned for panel B: pseudovirus + buffer control, pseudovirus + 100 nM OKT9, pseudovirus + 100 nM OKT9-Fab, and pseudovirus + 100 nM anti-EGFR as a nonrelevant antibody control. The reference bar indicates 50 μm. (B) Fluorescence microscopy of HEK-293T cells treated 48 h with buffer control, 100 nM OKT9, 100 nM OKT9-Fab, or 100 nM anti-EGFR, labeled with the DiO membrane dye (green), and incubated for 30 s with transferrin-TMR (red). The reference bar indicates 34 μm. (C) Flow cytometry dot plot analysis of JUNV, MACV, SABV-L, and LASV pseudovirus internalization in HEK-293T cells showing eGFP expression in the absence and presence of 100 nM OKT9, OKT9-Fab, and anti-EGFR. In the case of MACV, JUNV, and SABV-L, a reduction of the percent eGFP-positive events occurs in the presence of OKT9 and OKT9-Fab compared to buffer control and the nonrelevant antibody. In the case of LASV, similar levels of eGFP events are observed independently of the treatment. The percentages of eGFP-positive events are indicated inside the gates. (D) Inhibition of JUNV, MACV, and SABV-L pseudovirus internalization. Relative entry rate of JUNV, MACV, the recently reported SABV-L, and control LASV pseudovirus in HEK-293T cells was quantified in the presence of 100 nM OKT9-Fab, 100 nM OKT9, and 100 nM anti-EGFR. The data were 100% normalized with the cells without treatment, and the significant differences are indicated by comparing Fab OKT9, OKT9, and the negative-control anti-EGFR versus no treatment (*, *P* < 0.005, Student's *t* test for unpaired data of triplicate determinations). (E) Relative entry rate of pseudoviruses decorated with the GP1/GP2 complex of SABV, JUNV, MACV, GTOV, CHAV, SABV-L, and LASV to HEK-293T cells in the presence of OKT9 (0.01 to 100 nM). Pseudoviruses were loaded with an eGFP expression vector to express once internalized. After 48 h, the cells were fixed and the percentage of positive internalization events quantified by flow cytometry. The data are expressed as the means ± standard deviations (SD) from the sample. The data were normalized to 100% with the cells without treatment. (F) OKT9 and OKT9-Fab inhibit the infectivity of JUNV IV4454 virus strain. A549 cells were preincubated for 1 h with 200 nM OKT9, OKT9-Fab, anti-EGFR, or medium alone (control) and then infected with JUNV at an MOI of 0.01. After 1 h of incubation, viral inocula were replaced with the respective antibody-supplemented medium or medium alone, and 24 h postinfection, total JUNV production in A549 cell supernatants was measured using a PFU assay in Vero cells. The graph shows means ± SD from a representative experiment (from four independent experiments). The statistical analysis performed was ANOVA followed by Duncan’s test (**, *P* < 0.05). (G) Set of 200 nM OKT9-, OKT9-Fab-, anti-EGFR-, or medium alone (control)-treated A549 cells monolayers was harvested with TRIzol for RNA extraction 18 h postinfection with JUNV IV4454. Viral RNA (z gene) was quantified using RT-PCR, using actin as a housekeeping gene. The graph shows means ± SD from two independent experiments. The statistical analysis performed was ANOVA followed by Dunnett’s test (*, *P* < 0.0001).

Entry of pseudotyped viruses presenting clade B NWM glycoproteins into cells expressing hTfR1 was monitored through their expression of eGFP upon cellular entry, serving as a platform to assess the effectiveness of hTfR1 blockade by OKT9 (7). In this context, both OKT9 and OKT9-Fab inhibit the internalization of clade B NWM glycoprotein-presenting pseudovirus into HEK-293T cells. Unperturbed pseudovirus presenting glycoproteins from JUNV, SABV, MACV, GTOV, CHAV, SABV-L, and LASV all entered cells and produced green fluorescence. Cultured cells preincubated with 100 nM OKT9-Fab, OKT9, or a non-TfR1 specific antibody were exposed to JUNV, MACV, SABV-L, and LASV pseudovirus. Wide-field fluorescence images of cells treated with OKT9 or OKT9-Fab showed significantly lower levels of eGFP expression than cells without antibody treatment (Fig. 2A). While treatment with anti-TfR1 antibodies has been associated with downregulation of cell surface TfR1 (17), HEK-293T cells incubated with 100 mM OKT9 or OKT9-Fab for 48 h showed no decrease in cell binding of Tf-TMR. Fluorescence microscopy images showed similar binding of Tf to the cell surface across all conditions, as indicated by colocalization with the membrane-targeting fluorophore DiO (Fig. 2B). Changes in pseudovirus internalization were quantified by flow cytometry (Fig. 2C and D), showing decreased internalization of MACV, JUNV, and SABV-L in the presence of OKT9 and OKT9-Fab. Internalization of LASV pseudovirus was unaffected by OKT9 or OKT9-Fab, while a non-TfR1 specific antibody did not affect pseudovirus internalization (Fig. 2C and D). A 30-min preincubation of cells with OKT9 showed dose-dependent inhibition of pseudovirus internalization (Fig. 2E), yielding 50% inhibitory concentration (IC_50_) values of 0.329 nM (JUNV), 0.234 nM (SABV), 1.415 nM (MACV), 0.362 nM (GTOV), 0.274 nM (CHAV), and 0.632 nM (SABV-L) (Table 2).

**TABLE 2:** Analysis of NWM pseudovirus inhibition of internalization by OKT9

Parameter	SABV	JUNV	MACV	GTOV	SABV-L	CHAV
IC_50_^*a*^ (nM)	0.2339	0.3294	1.4150	0.3622	0.6320	0.2740
95% CI, IC_50_ (nM)	0.1393 to 0.3954	0.1019 to 0.9872	0.7233 to 2.865	0.2056 to 0.6384	0.4270 to 0.9354	0.2213 to 0.3399
*R* ^2^	0.9609	0.8658	0.9311	0.9434	0.9803	0.9948

a The fitting model used is *Y* = (bottom + top-bottom)/(1 + 10^(^*^X^*^-LogIC50)^), where IC_50_ is the concentration of OKT9 that gives a response halfway between bottom and top, and top and bottom are plateaus in the units of the *y* axis.

Both OKT9 and OKT9-Fab blocked entry of a replicating strain of JUNV into A549 cells. Viral replication was measured 24 h postinfection by quantifying viral particles released to the supernatants and by analyzing viral RNA levels in infected cell monolayers. Cultures treated with OKT9 or OKT9-Fab produced smaller amounts of viral particles, as determined by plaque assays (Fig. 2F), and also had lower levels of viral RNA, as measured by reverse transcription-quantitative PCR (RT-qPCR) (Fig. 2G), compared to the nontreated control. These results suggest that both OKT9 and OKT9-Fab blocked viral entry, reducing the cell susceptibility to this virus and, thus, preventing cell infection. The decrease in viral replication is specific, since cultures treated with an IgG that did not target TfR1 showed no significant differences from buffer control.

### The crystallographic structure of OKT9-Fab.

**FIG 3:**
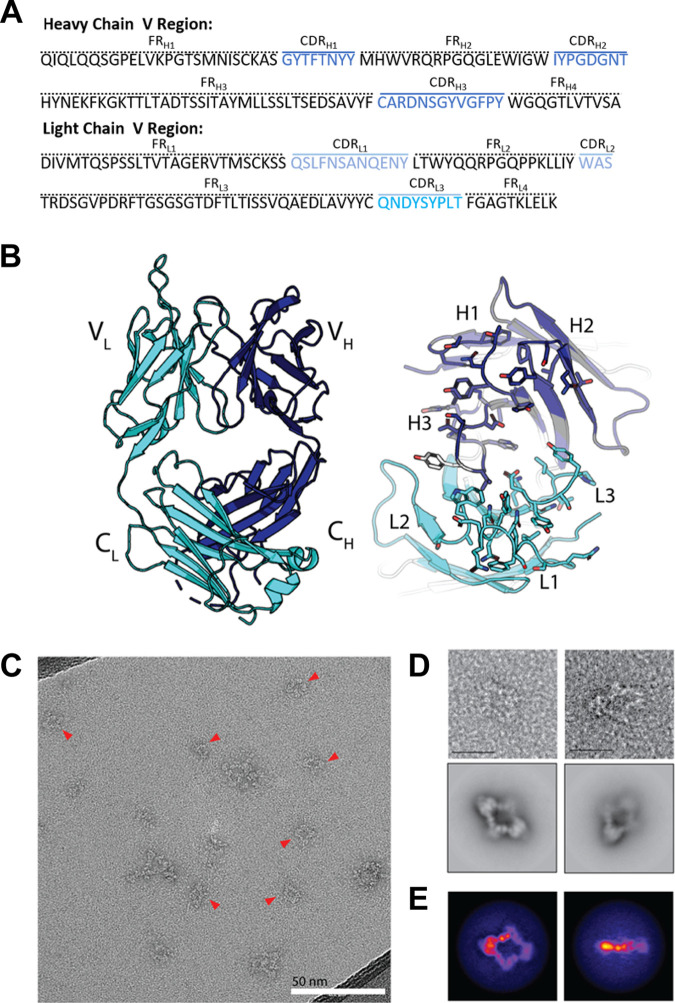
Crystallographic structure of OKT9-Fab and visualization of the hTfR1-OKT9 complex. (A) Amino acid sequence of OKT9 heavy-chain (top) and light-chain (bottom) variable region. In blue are the heavy-chain CDRs H1, H2, and H3 and in light blue the light-chain CDRs L1, L2, and L3. (B) X-ray crystallographic structure of OKT9-Fab with heavy chain colored in slate and light chain in cyan. Loops corresponding to variable light chain CDRs are labeled L1, L2, and L3, and CDRs for the variable heavy chain are labeled H1, H2, and H3, where unmodeled regions are shown as dashed lines. An unmodeled tyrosine residue in CDR_H3_ is shown in white. (C) Micrograph of negatively stained sample containing hTfR1-OKT9 complex. Red triangles indicate particles selected for 2D classification (scale bar, 50 nm). (D) Representative particles and 2D class averages of negatively stained hTfR1-OKT9 complexes on ultrathin carbon (scale bar, 200 Å). (E) Projections of 3D hTfR1-OKT9 complex density along two orthogonal directions. 3D density was obtained by *ab initio* reconstruction from negative-stain images of the complex.

A crystal structure of OKT9-Fab was determined to 2.0-Å resolution by microfocal X-ray diffraction (Table 3). The molecule crystallized with space group P2_1_2_1_2 with a single molecule in the asymmetric unit. The structure was determined by molecular replacement using chains from PDB entries 4Q0X and 4OTX. Sequences for the variable light and heavy chains, determined by sequencing of their genomic loci from the OKT9 hybridoma cell line (Fig. 3A), were used as guides during model building and data refinement (Fig. 3B).

**TABLE 3:** Data collection and refinement statistics for OKT9-Fab

Parameter	Value
Data collection	
Space group	P 2_1_ 2_1_ 2
Cell constants a, b, c (Å)	94.66, 113.96, 40.84
Cell constants α, β, γ (°)	90, 90, 90
Resolution (Å)	48.82–2.00
% Data completeness	99.2 (48.82–2.00)
Mean I/σ(I)	15.66 (3.12)
Wilson B-factor (Å^2^)	34.78
Refinement	
No. of total reflections	154,169
No. of unique reflections	30,478
CC_1/2_	99.9
No. of reflections used in refinement	30,471
No. of reflections used for Rfree	3,048
Rwork	0.2164
Rfree	0.2535
No. of nonhydrogen atoms	3,475
No. of protein residues	433
Wavelength (Å)	0.979
Ramachandran plot (%)	
Residues in favored region	97.65
Residues in allowed region	2.35
Residues in outlier region	0

The overall structure of OKT9-Fab resembles a canonical murine IgG1 variable region. All three complementary determining regions (CDRs) in the heavy and light chains were resolved (Fig. 3B). Their sequences were identified as CDR_L1_ QSLFNSANQENY (27-38), CDR_L2_ WAS (56-58), CDR_L3_ QNDYSYPLT (95-103), CDR_H1_ GYTFTNYY (26-33), CDR_H2_ IYPGDGNT (51-58), and CDR_H3_ ARDNSGYVGFPY (97-108). The constant heavy-chain region spanning from A136 to T139 showed poor density that precluded building during refinement and remained unbuilt in the final structure. Of greatest relevance was missing density for residue Y103 in CDR 3 of the heavy chain (Fig. 3B). Its side chain could not be placed during refinement and was omitted from our deposited structure but was included in subsequent computational analyses.

### OKT9 binds hTfR1 to form closed bivalent complexes identifiable by electron microscopy.

**FIG 4:**
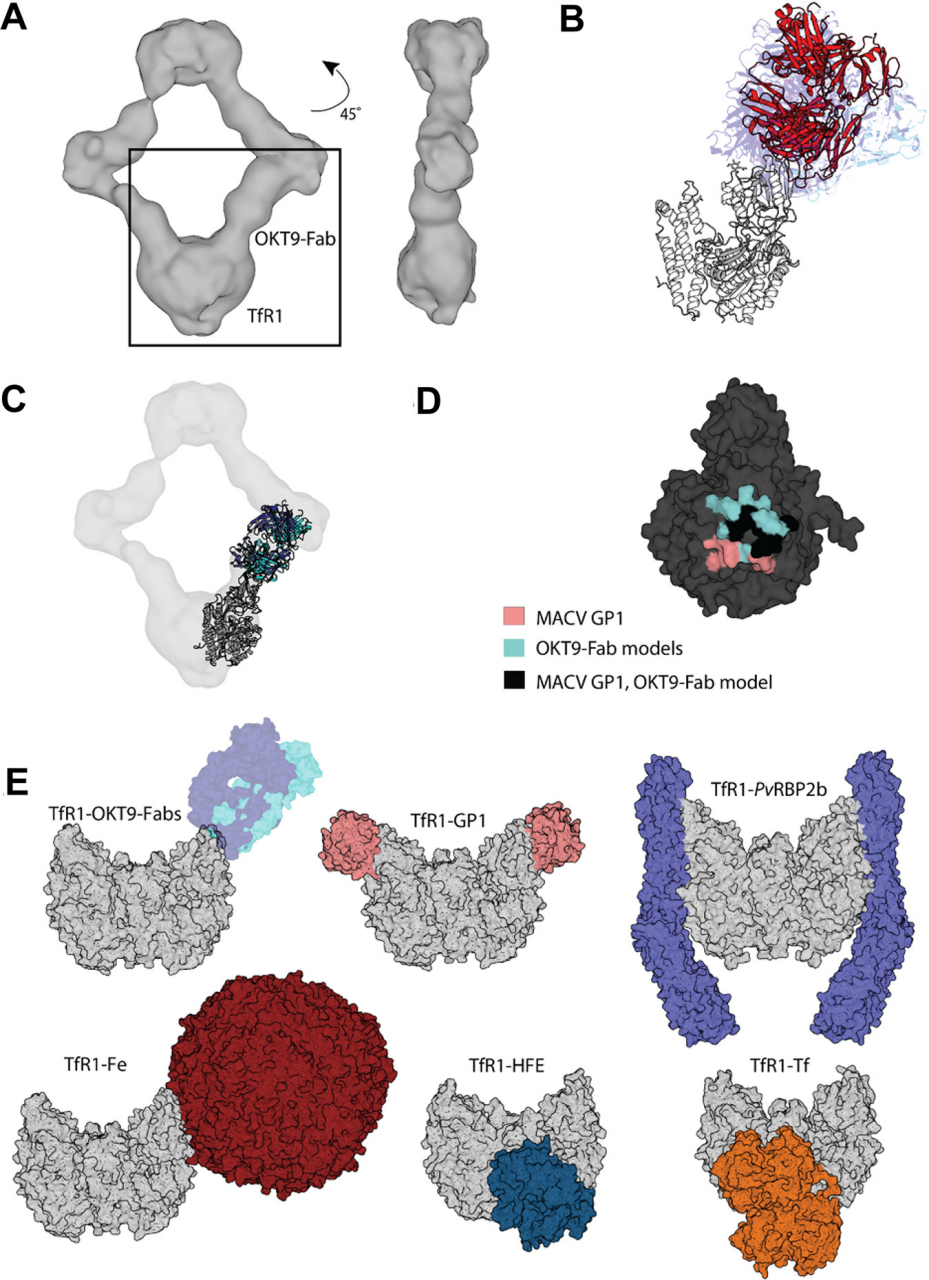
Docking model of hTfR1-OKT9 complex and relationship to natural ligands and human pathogen molecular ligands. (A) Isosurface rendering of hTfR1-OKT9 3D density map with an estimated resolution of 11 Å. Inset shows a well-resolved region of the 3D density, corresponding to a monomeric transferrin receptor bound by an OKT9-Fab. Front (left) and side (right) views are shown. (B) Front views of all 10 ClusPro-generated models using OKT9-Fab and 3KAS hTfR1 apical domain monomer (white). Best-fit models used for analysis are highlighted in red; other models are shown in hues of blue. (C) Overlay of ClusPro generated model using OKT9-Fab and crystal structure PDB entry 3KAS with hTfR1-OKT9 3D density. (D) Surface representation of the 3KAS apical domain (dark gray) colored to illustrate the binding interface with MACV GP1 protein (salmon), OKT9-Fab model (blue), or both (black). (E, top) Transferrin receptor (gray) bound to natural ligands (transferrin, PDB entry 1SUV; hereditary hemochromatosis factor [HFE] [43], PDB entry 1DE4; and ferritin, PDB entry 6GSR). (Bottom) Transferrin receptor bound to OKT9-Fabs and human pathogens (MACV-GP1, PDB entry 3KAS; *P. vivax*, PDB entry 6D04).

To evaluate the basis for inhibition of cellular internalization of NWM by OKT9 targeting of hTfR1, we sought to reveal the mode of interaction between full-length OKT9 and hTfR1. A solution containing OKT9 and hTfR1 was drop-cast onto electron microscopy grids and negatively stained with uranyl acetate. Use of an equimolar ratio of OKT9 to hTfR1 allowed for the formation of stable, homogenous assemblies; complexes with a prominent diamond-shaped structure were identified (Fig. 3C). Imaging of these grids at various tilt angles, ranging from 0 to 45 degrees, circumvented the preferred orientation of the flat, diamond-like assemblies on the grid. Combining images across all tilts revealed multimeric complexes of receptor and OKT9 that could be subclassified using single-particle methods (Fig. 3C). A low-resolution structure of OKT9 in complex with hTfR1 was determined from these images. Particles contributing to the best classes were used to generate an *ab initio* model used for further refinement (Fig. 3E). Despite low particle count and preferred particle orientations, the refined three-dimensional (3D) density determined from these data revealed the binding interface between hTfR1 and OKT9 (Fig. 4A). The Fc region of OKT9 could not be identified in 2D averages or in the final 3D density, likely due to the flexibility of the hinge region (Fig. 3C). The resulting density showed sufficient resolution to suggest the relative orientation of the OKT9 variable regions with respect to the apical domain of hTfR1.

### Computational prediction of OKT9-Fab binding footprint on hTfR1.

We validated the configuration of OKT9 relative to hTfR1 in our reconstruction by comparing the reconstructed density to a series of docked models generated using the web-based server ClusPro 2.0 (18). For this, we relied on the known structure of hTfR1 (PDB entry 3KAS) and our crystal structure of OKT9-Fab. We used ClusPro in antibody mode, where CDR residues and the framework surface residues were excluded in the repulsion mask of the Fab. An attraction mask was generated on the surface residues of hTfR1 based on the reconstructed 3D density of the OKT9-TfR1 complex. Lastly, a repulsion mask was generated to exclude other residues on hTfR1 that flanked this area. ClusPro generated 10 docked models (Fig. 4B). OKT9-Fab models docked onto hTfR1 were fit onto the 3D density maps for the OKT9-hTfR1 complex using a rigid-body procedure in Chimera. This procedure yielded two top models that matched the relative orientation of 3D density for OKT9-Fab with respect to hTfR1 and best fit the 3D antibody density (Fig. 4C). While the resolution of this 3D model does not allow for the identification of residue-level interactions, the overall binding footprint of OKT9 on hTfR1 is identifiable (Fig. 4D). Analysis of the putative binding interface for the best of these models shows a significant overlap of the binding site for MACV GP1 on hTfR1, with both OKT9 and MACV GP1 competing to bind an exposed loop in the hTfR1 apical domain (Fig. 4E).

## DISCUSSION

The treatment of infectious diseases caused by viral agents with high sequence and structural diversity presents a challenge for targeted therapy (19). Broadly neutralizing antiviral antibodies are scarce given the sequence diversity of viral epitopes and their constant evolutionary pressure; this applies to NWMs. In contrast, strategies that target a host receptor, such as hTfR1, are appealing given its cell surface accessibility, low sequence variability across populations, and well-characterized structure and physiology (20). The identification of a single conserved epitope on hTfR1 as a shared binding site for clade B NWMs and the determination of a crystallographic structure of MACV GP1 bound to the conserved epitope have illuminated avenues for NWM neutralization. This knowledge has already enabled the isolation and characterization of various NWM neutralizing antibodies (4, 6, 7, 21).

Antibodies capable of neutralizing or preventing cell entry of more than one NWM have targeted either the site of hTfR1 binding on MACV and JUNV GP1 or the hTfR1 apical domain itself (9, 22). Taking advantage of this opportunity for broad NWM neutralization by hTfR1 targeting (8), we establish the well-known, hTfR1-targeting murine monoclonal antibody OKT9 as a framework for future development of NWM-neutralizing agents that reduce cellular uptake of NWMs. OKT9 competes with only one known natural ligand, heavy-chain ferritin (Fe) (Fig. 4E) (23). However, homeostatic alterations resulting from this competition are unclear given that iron uptake via ferritin is not a principal mechanism of iron uptake into cells. Importantly, OKT9 is not expected to interfere with transferrin (Tf) binding to hTfR1, as that interaction relies entirely on the membrane, proximal helical, and protease-like domains (Fig. 4E) (20). Moreover, despite previous reports of anti-TfR1 antibodies depleting cell surface TfR1 by altering its recycling and inducing its accumulation and degradation in late endosomes or lysosomes (17), 48-h treatment of HEK-293T cells with OKT9 or OKT9-Fab did not significantly alter cell surface binding of Tf.

The variable region of OKT9 binds hTfR1 with nanomolar affinity, allowing full-length OKT9 to inhibit cellular entry by NWM with nanomolar IC_50_. This efficient inhibition of NWM internalization is enabled by the high overall avidity of full-length OKT9, with an EC_50_ value an order of magnitude better than that for OKT9-Fab. The affinity estimated for the binding of OKT9-Fab to hTfR1 is on par with that previously reported for other TfR1-binding antibodies, including ch128.1, with equilibrium dissociation constant (*K_D_*) values of 4.82 nM and 5.7 nM, respectively (9). OKT9-Fab also binds hTfR1 with equal or greater affinity than virus-neutralizing antibody variable fragments targeting GP1 of JUNV (5 nM) and MACV (16.3 nM) (22). These comparisons illustrate the opportunity for high-affinity inhibition of viral entry by targeting hTfR1, provided the targeting agent effectively precludes binding of NWM GP1 to the receptor. The potency of OKT9 viral entry inhibition is further demonstrated on a competent attenuated JUNV, where treatment with the antibody led to reduced plaque formation and viral detection by RT-PCR. This approach succeeded despite the potential for non-TfR1-mediated viral entry, possibly involving C-type lectins DC-SIGN/L-SIGN (24, 25), the phosphatidylserine receptors Axl (26), TIM-1 (27), or fluid-phase uptake.

Use of single variable-domain antibodies or antibody fragments is warranted when potent enough to act within the timescale of their half-life. Antibody fragments are especially preferable when their corresponding full-length antibody is associated with a risk of immunotoxic effects in humans (28). For these reasons, use of OKT9-Fab could be favored over its full-length counterpart given a history of potential homeostatic dysregulation by full-length OKT9: incubation of the erythroleukemia cell line K562 to full-length OKT9 induces cellular redistribution and increased degradation of TfR1 (29). Potential autoimmune reactions add to concerns over homeostatic dysregulation induced by full-length OKT9, as with other full-length antibodies. The superior avidity of full-length OKT9 over OKT9-Fab can also be a detriment, as has been observed in relation to transcytosis across the blood-brain barrier (30). These concerns are especially pressing for hTfR1 targeting given its ubiquitous expression across tissue types. However, several of these potential issues can be mitigated by engineering of full-length antibodies to suppress binding of immune effector molecules to their Fc region and/or introducing mutations that reduce the affinity of each variable region.

The overall site of interaction between the OKT9 variable region and the apical domain of hTfR1 stands near a previously identified loop thought to be a key determinant for MACV GP1 binding on hTfR1. In fact, based on negative-stain single-particle electron microscopy and computational docking models, residues D204, Y211, and N348 on hTfR1 are hypothesized to be bound by both MACV GP1 and OKT9 and are key determinants of zoonotic transmission across arenaviruses (31). Direct competitive inhibition of NWM GP1 binding to hTfR1 by the OKT9-Fab is supported by biolayer interferometry-based competition assays and presents a molecular framework for dissecting NWM blockade by hTfR1-targeting antibodies. Therefore, hTfR1 blockade by the OKT9 variable region alone is sufficient for blocking viral entry and permits the discovery of new NWM viruses that utilize TfR1 as a port of entry into human cells. We demonstrate this by investigating the recently reported and lethal Sabia variant (SABV-L), whose genome sequence has been determined, and its S segments coding for its glycoproteins share 87% homology with those of SABV strain SPH114202 (14). OKT9 blockade of GP-mediated cellular entry of this new virus not only confirms its use of hTfR1 as a portal of entry into human cells but also could inform on the impact of hTfR1 binding by other pathogens, such as the malaria parasite Plasmodium vivax (Fig. 4E) (32). Importantly, the unique geometry of the ternary complex formed by dimeric receptor and OKT9 in solution suggest efficient steric occlusion of the hTfR1 apical domain with limited cross-linking at the cell surface.

Monoclonal antibody-based therapies are now front-line treatments for a variety of diseases. Only four monoclonal antibodies have been FDA approved to treat an infectious disease. Three of these directly target infectious pathogens, including palivizumab for respiratory syncytial virus prophylaxis (46) and raxibacumab and obiltoxaximab for treatment of inhalational anthrax (33, 34). In contrast, ibalizumab targets CD4 to block the entry of HIV-1 into human cells (35). Because of the rare occurrence of NWHFs as well as their relatively short manifestation in humans, broadly neutralizing antibodies represent feasible antiviral therapeutics for existing and emerging clade B NWMs. Our approach further enables a possible route to treatment for those infected or at risk of getting infected by hemorrhagic fever viruses that share hTfR1 as a host receptor, including viral strains that are yet to be identified.

## MATERIALS AND METHODS

### OKT9 IgG1 production.

Murine hybridomas expressing the monoclonal antibody OKT9 were obtained from the ATCC (catalog number CRL-8021). Cultures initially grew in Iscove’s modified Dulbecco’s medium (IMDM) supplemented with fetal bovine serum (FBS) (10%), GlutaMAX, and penicillin-streptomycin. Once sufficiently proliferated, the cells were passed to 2-liter roller bottles and weaned from FBS dependence using increasing volumes of SFM (Gibco, USA) before the medium was harvested. Cells were spun down, and the supernatant was filtered by gravity filtration against cellulose paper (Millipore, USA) to remove any large particulate matter. This was then passed through a 0.22-μm Millipore filter, and the clarified medium was purified by affinity chromatography using a protein G column HiTrap protein G HP (GE LifeSciences, USA) equilibrated to Tris-buffered saline (TBS) with 200 mM NaCl at pH 7.4. The OKT9 was eluted using a gradient of TBS with 100 mM glycine and 200 mM NaCl at pH 2.6; 100 μl of 1 M TBS at pH 9.0 was added to each fraction. These fractions were analyzed by SDS-PAGE, and the purest samples were consolidated and concentrated to ∼200 to 500 μl using an Amicon Ultra-4 10K centrifugal filter device (Millipore, USA). The resulting protein was further purified via SEC using an Enrich SEC 650 10- by 300-mm column (Bio-Rad, USA) connected to an NGC Quest 100 chromatography system instrument (Bio-Rad, USA) equilibrated with TBS. SEC fractions were analyzed by SDS-PAGE and the purest bands were concentrated using an Amicon Ultra-4 10K centrifugal filter device to ∼500 μl.

### OKT9-Fab production.

Purified OKT9 was digested with ficin using the mouse IgG_1_ Fab and F(ab)_2_ preparation kit (Pierce, Thermo Scientific, USA) according to the manufacturer’s instructions. After digestion, the sample was purified by SEC using an Enrich SEC 650 10- by 300-mm column (Bio-Rad, USA) and NGC Quest 100 chromatography system chromatography equipment (Bio-Rad, USA) equilibrated with TBS. These fractions were collected and analyzed by SDS-PAGE to confirm purity and integrity. Fractions were subsequently concentrated to ∼100 μl using an Amicon Ultra-4 10K centrifugal filter device for use in crystallization trays.

### OKT9 variable region sequence analysis.

OKT9 heavy- and light-chain variable region residue compositions were determined using mRNA isolated from the hybridoma cell line subjected to RT-PCR with mouse-specific primer sets to amplify the target regions by using a proprietary sequencing procedure (LakePharma, USA).

### Production and purification of human sTfR1.

Baby hamster kidney (BHK) cells expressing the histidine-tagged soluble extracellular domain of human TfR1 (sTfR1) were cultured at 37°C in IMDM with 1× GlutaMAX and 1× penicillin-streptomycin antibiotic mixture and certified 10% fetal bovine serum (Thermo Fisher, USA). BHK cells were trypsinized with TrypLE express (Thermo Fisher, USA), centrifuged, and replated after harvest of the sTfR1-containing supernatant. Supernatant was clarified with a 0.22-μm Millipore vacuum filter and passed through a His trap (GE Healthcare, Millipore, USA) column via fast protein liquid chromatography (FPLC) on a BioLogicDuoFlow (Bio-Rad, USA). The column was washed with a 90:10 mixture of TBS (250 mM NaCl, 20 mM Tris, pH 7.4) and imidazole buffers (250 mM NaCl, 20 mM Tris, 500 mM imidazole) and then eluted via a gradient of increasing imidazole in TBS. Elution fractions were analyzed via SDS-PAGE, and similar samples were pooled and further purified via an Enrich SEC 650 10- by 300-mm column (Bio-Rad, USA) and NGC Quest 100 chromatography system chromatography equipment (Bio-Rad, USA).

### Size exclusion chromatography of OKT9-Fab alone and in complex with soluble human TfR1.

Three hundred micrograms of OKT9-Fab in 500 μl of TBS (50 mM Tris, 150 mM NaCl, pH 7.6) was injected into a 2-ml loop of a medium-pressure liquid chromatography system (NGC; Bio-Rad, USA) and run through an Enrich SEC 650 column (Bio-Rad, USA) using TBS at a flow rate of 0.8 ml/min. A similar procedure was performed using 900 μg of sTfR1 alone. A complex of sTfR1 and OKT9-Fab was allowed to form by mixing their solutions at a 3:1 molar ratio of receptor to Fab, followed by incubation at room temperature for 20 min prior to loading into the injection loop. All fractions were collected and analyzed by SDS-PAGE: 15 μl of each sample was added to 3.5 μl of NuPAGE LDS sample buffer, heated to 98°C for 10 min before being loaded into a precast NuPAGE 4 to 12% bis-acrylamide gel; sizing was analyzed against a See Blue 2 protein standard. Samples were run for 1 h at 100 V and then stained with SimplyBlue SafeStain before being imaged on an AzureBiosystems 300c gel imager using the AzureBiosystems gel analysis software. Analysis of gel images was performed in ImageJ.

### Binding of OKT9 to human sTfR1 by ELISA.

A 96-well plate was covered with 50 μl/well of a 1.5-μg/ml solution of human sTfR1 in carbonate buffer (0.015 M Na_2_CO_3_, 0.035 M NaHCO_3_, pH 9.3) for 16 h at 4°C. The plate was washed 3 times with 200 μl/well phosphate-buffered saline (PBS), 0.05% Tween 20; 200 μl of blocking solution (5% skim milk powder, 1% bovine serum albumin (BSA), and 0.02% sodium azide in PBS, pH 7.4) was added to each well and incubated for 1 h at room temperature. The plate was then washed 3 times with 200 μl/well of PBS, 0.05% Tween 20, and 50 μl/well of OKT9 (0.025 to 25.8 nM) or OKT9-Fab (0.077 to 76.9 nM) was added to the wells and incubated for 2 h at room temperature. At the end of the incubation time, the plate was washed 3 times with 200 μl/well of PBS, 0.05% Tween 20 and incubated with an anti-mouse IgG horseradish peroxidase (Vector Laboratories) (1:2,000) in a 1:3 dilution of blocking solution for 1 h at 37°C. The plate was then washed 4 times with 200 μl/well of PBS, 0.05% Tween 20 and 80 μl/well of TMB substrate solution (BD Biosciences) was added and incubated for 30 to 45 min at room temperature. The reaction was stopped with 80 μl/well of 2 M H_2_SO_4_, and the plate was read by absorbance at an optical density at 450 nm with an enzyme-linked immunosorbent assay (ELISA) plate reader (Thermo Scientific, USA). EC_50_ values were obtained by nonlinear regression fitting to a variable slope, four-parameter dose-response model using GraphPad Prism 8 (www.graphpad.com).

### Binding analysis of OKT9 to human sTfR1 by BLI.

All BLI assays were performed on a ForteBio Octet 96RED at a volume for all the solutions of 200 μl/well, with agitation set to 1,000 rpm at 30°C in solid black 96-well plates (Greiner, USA). HIS1K (Anti-Penta-HIS, USA) probes were equilibrated for 600 s in TBS, pH 7.6 (50 mM Tris-Cl, pH 7.6, 150 mM NaCl), prior to sample loading; 100 μg/ml sTfR1-His ligand was loaded for 60 s, followed by a biosensor baseline equilibration step in TBS for 60 s. Typical capture levels were between 0.4 and 0.5 nm and variability within a row of eight tips did not exceed 0.1 nm. Loading was followed by a 60-s association step, during which probes were exposed to OKT9-Fab at concentrations of 250 nM, 125 nM, and 62.5 nM in TBS. Dissociation of Fab was performed in TBS for 900 s. A similar experiment was performed in which 2-fold dilutions from 857.1 μg/ml to 13.4 μg/ml sTfR1-His were immobilized to HIS1K probes for 90 s, followed by a baseline equilibration step in TBS for 90 s. Association of OKT9-Fab at 70 nM for 60 s was next, followed by dissociation in TBS buffer for 600 s.

Data analysis and curve fitting were conducted using Octet 96RED analysis software. Correction of any systematic baseline drift was achieved by subtracting the shift recorded for a reference sensor loaded without sTfR1-His and incubated with OKT9-Fab. All data were filtered using the Savitzky-Golay algorithm and fitted with binding equations that assumed a 1:1 interaction. Global nonlinear least-squares fitting was performed on data sets that included association and dissociation steps.

### MACV GP1-Fc BLI competition assay.

The soluble human transferrin receptor 1 (sTfR1) for these studies was expressed in HEK293S cells. The ectodomain of human transferrin receptor 1 (GenBank accession no. NM_003234.3, residues 121 to 760) was cloned into the pHLsec expression vector downstream of the pHLsec (36) secretion signal. sTfR1 was transfected in HEK293S GnTI^−/−^ cells (ATCC CRL-3022TM) using linear polyethylenimine (PEI). HEK293S GnTI^−/−^ cells were cultured as suspension cells in FreeStyle 293 expression medium (Gibco, Thermo Scientific, USA) supplemented with 2% (vol/vol) ultralow IgG FBS (Thermo Fisher, USA) and penicillin-streptomycin. Supernatant was harvested 72 h posttransfection. sTfR1 was purified by affinity chromatography using human transferrin-coupled Sepharose as previously described (8, 37, 38) and eluted in buffer containing 2 M potassium chloride and 50 mM HEPES (pH 7.5). sTfR1 was further purified by size-exclusion chromatography on a Superdex 200 increase column (GE Healthcare Life Sciences, USA), eluting at the expected retention time. DNA encoding the MACV GP1 subunit (GenBank accession no. NC_005078, residues 87 to 250) was cloned into a pVRC8400 vector containing human IgG Fc (a gift from Aaron Schmidt). The resulting vector was transfected in HEK293T cells grown in suspension. The MACV GP1-Fc fusion protein was purified by protein A affinity purification according to the manufacturer’s protocol (Thermo Fisher Scientific), followed by size-exclusion chromatography on a Superdex 200 increase column. The assay was performed using an Octet RED96 system (ForteBio, USA). MACV GP1-Fc was loaded onto anti-human IgG Fc capture biosensor tips (ForteBio, USA) at 17 nM for 100 s in kinetic buffer (PBS containing 0.02% [vol/vol] Tween and 0.1% BSA), followed by a baseline measurement taken for 120 s. Next, sTfR1 at 1.5 μM, sTfR1 at 1.5 μM plus OKT-9 at 2 μM, or sTfR1 at 1.5 μM plus transferrin at 2 μM were associated for 300 s. The sensor tip was then placed in kinetic buffer for 300 s for a subsequent dissociation step.

### Generation of pseudotyped virus presenting NWHF glycoproteins.

The procedures and vectors used to make the pseudoviruses used here were described previously (7). Briefly, pseudotyped virus particles presenting the surface glycoproteins of JUNV, MACV, CHAV, GTOV, SABV, SABV-L, and LASV were generated by cotransfecting HEK-293T cells with vectors for the expression of the polyprotein gag/pol of the nonreplicating murine leukemia virus, GP1 and GP2 of each of the viruses, and the retroviral vector pQCXIX expressing eGFP (enhanced green fluorescent protein). Transfection was achieved by mixing equimolar quantities of all three vectors in the presence of calcium phosphate (7) or Lipofectamine 2000 (Invitrogen, USA). HEK-293T cells were incubated with the vector cocktail at 37°C with 5% CO_2_. Twenty-four hours posttransfection, medium was exchanged, and supernatant with the released pseudoviruses was collected 48 h and 72 h posttransfection. The medium containing each of the pseudoviruses was clarified by centrifugation at 10,000 × *g* and subsequently filtered with a 0.45-μm polyvinylidene difluoride filter (Jetbiofil, China), and pseudovirus particles were further concentrated using a Vivaspin 1,000-kDa filter (Sartorius, USA). Aliquots were used immediately or stored at −80°C.

### Cellular internalization of pseudotyped virus presenting NWHF glycoproteins.

HEK-293T cells at approximately 40% confluence were preincubated with medium containing a serial dilution (from 0.001 to 100 nM) of OKT9 or with 100 nM OKT9-Fab and 50 nM anti-EGFR (negative control) for 30 min at 37°C with 5% CO_2_ in 48-well plates (CellATTACH Biofil, China). One hundred microliters of the concentrated solution of pseudovirus particles of JUNV, MACV, CHAV, GTOV, SABV, SABV-L, and LASV (control) in the presence of the different antibody conditions was added to each well. Cells were incubated for 16 h and washed with fresh medium. After 48 h of incubation at 37°C with 5% CO_2_, cells were inspected by wide-field fluorescence microscopy on an Olympus CKX41 inverted microscope (Olympus, Japan) equipped with an LCAch N 20×/0.40 Php objective (Olympus, Japan), and bright-field and fluorescence images were acquired with a Q-Color5 digital imaging system (Olympus, Japan). Cells were then fixed with 2% paraformaldehyde, and the inhibition of internalization was quantified by flow cytometry using a FACScanto (BD Biosciences, USA) equipped with an argon ion laser (488 nm), with filter settings for GFP (530/30 nm), and the flow data were analyzed with the software FlowJo 7.6 (BD Biosciences, USA). Relative internalization was determined as the percentage of cells positive for GFP normalized to cells treated in the absence of antibodies. The IC_50_ data were calculated from each pseudovirus dose-response data set. For this analysis, we used a dose-response model assuming that the log (inhibitor) versus response curves follow a symmetrical sigmoidal shape. The IC_50_ of OKT9 for JUNV, MACV, CHAV, GTOV, SABV, and SABV-L was determined as the concentration that provokes a response halfway between the maximal (top) response and the maximally inhibited (bottom) response. We did not include the estimated IC_50_ of LASV, because it did not exhibit inhibition in the OKT9 tested concentration range. The fitting model used is *Y* = bottom + top-bottom)/(1 + 10^(^*^X^*^-LogIC50)^), where IC_50_ is the concentration of OKT9 that gives a response halfway between bottom and top; and top and bottom are plateaus in the units of the *Y* axis. Statistical differences between OKT9, OKT9-Fab, and anti-EGFR compared to nontreated control conditions were analyzed using nonpaired *t* test with two tails and assuming different variances between groups.

### Cell labeling with fluorescent transferrin and a fluorescent membrane probe.

The fluorescent labeling of HEK-293T cells with Tf-TMR (Invitrogen, USA) and the lipophilic fluorophore Vybrant DiO cell-labeling solution (Invitrogen, USA) was performed by following the manufacturer's instructions. Briefly, HEK-293T cells at 50% confluence and grown in coverslips were incubated with medium containing 100 nM OKT9, 100 nM OKT9-Fab, 100 nM anti-EGFR, or no antibody for 48 h. Medium was exchanged, and 350 μl of 50 μg/ml Tf-TMR (red) diluted in medium without serum was added to each well. Cells were incubated 30 s and washed one time with PBS. After that, cells were fixed with 4% paraformaldehyde for 20 min. Cells were washed 3 times for 3 min with PBS, and 350 μl of Vybrant DiO (green) cell-labeling solution diluted 1:200 was added and incubated for 20 min at 37°C. Next, cells were washed with PBS and mounted on a slide. Cells were inspected by wide-field fluorescence microscopy on an Olympus IX83 motorized confocal DSU (disk spinning unit) module (Olympus, Japan) equipped with a PlanAPO N 60×/1.42 objective. Bright-field and fluorescence images were acquired with ORCA FLASH 4.0 V2 digital CMOS (4 MP, 16 bits) (Hamamatsu, Japan) and CellSens Dimensions software (Olympus, Japan). The green and red image overlay was generated using the ImageJ Merge function.

### *In vitro* inhibition of JUNV infection.

A549 cells (ATCC CCL-185) were plated in 24-well plates at a density of 10^5^ cells per well. After 24 h, the cells were incubated for 1 h with 200 nM OKT9, OKT9-Fab, or a control IgG (anti-EGFR) or medium alone (control). After incubation, cells were infected for 1 h with JUNV IV4544 viral strain (15) at a multiplicity of infection (MOI) of 0.01 in the presence of antibodies. After removing the inoculum, cell monolayers were incubated with the respective antibody-supplemented medium or medium alone. The supernatants were harvested 24 h postinfection, and JUNV production was titrated by a standard plaque assay in Vero cells.

Quantitative real-time PCR determination of viral load was assessed as follows. A549 monolayers were harvested, and RNA was extracted using Tri Reagent (Molecular Research Center, USA) according to the manufacturer’s instructions. cDNA then was generated by use of murine reverse transcriptase M-MLV (Promega, USA) and random primers (Biodynamics, Argentina). This cDNA was amplified by real-time PCR using SYBR green complete mix detection (Roche, USA). Real-time PCR was carried out with an initial incubation at 95°C during 5 min, followed by 45 cycles of 30 s at 95°C, 45 s at 60°C, and 30 s at 72°C and a final step of 5 min at 72°C. Amplification plots were analyzed with Bio-Rad software, and the comparative threshold cycle method was used to determine viral gene expression relative to the β-actin cellular gene. Primer sequences are the following: Actin Fw, 5′-GAGACCTTCAACACCCCAGCC-3′; Actin Rv, 5′-GGCCATCTCTTGCTCGAAGTC-3′; Z Fw, 5′-ATGGGCAACTGCAACGGGGCATC-3′; Z Rv, 5′-CTATGGTGGTGGTGCTGTTGGCT-3′.

Statistical analysis was carried out using InfoStat software (http://www.infostat.com.ar). Randomized block analysis of variance (ANOVA) was performed in titration assays, while ANOVA was performed in RNA quantification assays. In both, statistical significance was assessed at a level of a *P* value of <0.05, followed by Dunnett’s or Duncan’s *post hoc* test (InfoStat; http://www.infostat.com.ar). Graphs were created using GraphPad Prism software (La Jolla, CA; www.graphpad.com).

### Crystallographic analysis of the OKT9-Fab.

The OKT9-Fab was concentrated to ∼10 mg/ml in 50 mM Tris-HCl, pH 7.5, with an Amicon Ultra-4 10K centrifugal filter device (Millipore, USA) and then filtered using a 0.22-μm centrifuge filter (Corning, USA) (Table 3). A LabTech mosquito crystal nanodispenser TTP robot (TTP LabTech, UK) was used to evaluate 576 different conditions in a pendant drop vapor diffusion system. The plates were incubated in an environment without vibrations at approximately 18°C. Several conditions produced crystals of various qualities, and a MgCl_2_-containing mother liquor was chosen for optimization. In a 24-well optimization plate (Hampton Research, USA), vapor diffusion/hanging-drop conditions in 1:1 and 1:2 drops of protein to mother liquor were prepared using various concentrations of Tris, MgCl_2_, and polyethylene glycol (PEG) 8000. Large, blade-like crystals grew under conditions containing 100 mM Tris, 200 mM MgCl_2_, and 20%, wt/vol, PEG 8000. These crystals were collected and passed through glycerol as a cryoprotectant, mounted on a microloop, and flash frozen into liquid nitrogen for transport. X-ray diffraction data were obtained at beamline 24-ID-E at the APS synchrotron (Advanced Photon Source, Argonne National Laboratory). Diffraction patterns were reduced in XDS and then merged and scaled in XSCALE (39). The structure was phased by molecular replacement using PHASER in the PHENIX suite of crystallographic software (40) with a chimeric model generated from two antibody Fabs (PDB entries 4Q0X and 4OTX). Following initial refinement in BUSTER (https://www.globalphasing.com/), final refinement was carried out in PHENIX, with model building in COOT (41). Visualization of models and structural analyses were performed in PyMOL (https://pymol.org/2/).

### Electron microscopy of OKT9-sTfR1 complexes.

Purified OKT9 and sTfR1 were diluted to 10 μg/ml and 2 μg/ml, respectively. A 1:1 ratio of diluted OKT9 and sTfR1 was then mixed and further diluted by a factor of two. OKT9-sTfR1 negative-stained grids were prepared for electron microscopy by placing a 2.5-μl drop of complex onto a glow-discharged ultrathin C film on Lacey Carbon 300 mesh (Ted Pella, Inc., USA) and washed twice with 2% uranyl acetate. Negative-stain grids were imaged at room temperature using a Tecnai F30 operating at 300 keV at a magnification of ×120,000 on a TVIPS XF-416 camera with a pixel size of 16 μm and a sensor with 4,096 by 4,096 pixels. These settings corresponded to a pixel size of approximately 2.0 Å/pixel. A total of 5 data sets were collected at various tilt angles to facilitate views of the complex and avoid orientation bias. In addition to zero tilt, data were collected at a tilt of 10, 25, 35, and 45°.

### Single-particle analysis of OKT9-sTfR1 complexes.

Global contrast transfer function was calculated using Gctf implemented in RELION 3.0.7 for micrographs in all data sets. Approximately 982 (0° tilt), 946 (10° tilt), 1,389 (25° tilt), 2,044 (35° tilt), and 909 (45° tilt) particles were manually picked from the respective data sets. OKT9-sTfR1 particles were extracted in RELION using a 250-pixel box size. After extraction, STAR files were joined to combine particles from all data sets. Particles were initially classified using *K* = 20 classes and angular sampling of 5°. Several rounds of 2D classification allowed for removal of poorly aligning particles. Final 2D classifications were performed using *K* = 4 with a final particle count of 4,191. RELION extracted particles were also imported into cryoSPARC 2.12.2 for 2D classification. Selected 2D classes containing 5,217 particles were used as inputs for an *ab initio* reconstruction. The resulting best class and volume containing 3,436 particles were used for homogenous refinement.

### ClusPro model generation.

The docking model of OKT9-Fab to hTfR1 was performed using the web server ClusPro docking algorithm (18). This software runs a rigid body docking, followed by a root mean standard deviations clustering ranked by the number of structures with the lowest energy and refined by energy minimization to remove steric clashes (18). The structures of OKT9-Fab and hTfR1 were uploaded to the ClusPro v2.0 server in antibody-antigen mode for docking (42). The hTfR1 monomeric structure extracted from the PDB entry 3KAS was defined as the ligand, while the newly determined structure of OKT9-Fab with a modeled Y103 side chain was defined as the receptor. To minimize the search region for docking, we relied on the 3D density of the complex of OKT9-hTfR1. Based on this density, we identified the overall region of the hTfR1 apical domain bound by OKT9. We selected an attraction mask that included hTfR1 surface residues D204-L212, N348-E350, S368-K371, and K374. We also applied a repulsion mask surrounding this interaction area on the hTfR1 apical domain that included the following surface residues: V228-L232, Y247-G252, I277, A340-E343, G351, D352, and M365-T367. For OKT9-Fab, we used a repulsion mask that excluded the surface residues of the heavy- and light-chain CDRs and the surface residues surrounding them. The repulsion mask included light-chain residues D1-C23, L39-K51, L53, I54, G63-G72, T78-C94, and G105-E219 and the heavy-chain residues Q1-A24, M34, W36-E46, I48, G49, T58, Y60-S76, T78-A97, and W109-R219. Residues in the attraction and repulsion masks were identified using PyMOL.

### Fit of ClusPro models into 3D density for OKT9-hTfR1 complex.

3D density corresponding to the final homogenous refinement map was loaded onto Chimera for further analysis. The ectodomain of the dimeric human transferrin crystal structure (PDB entry 1CX8) was manually placed within the map density, followed by the fit-in-map function to finalize placement of the structure. Monomeric hTfR1 (PDB entry 3KAS) and ClusPro inputs were placed using MatchMaker, given the dimer structure as a reference. All generated ClusPro models were subsequently fit using MatchMaker and 3KAS as a reference and were later manually inspected for fit onto the 3D density.

### Data availability.

The coordinates for the structure of OKT9-Fab have been deposited in the Worldwide Protein Data Bank (wwPDB) with accession code 6WX1.
