# Correction for Ferrero et al., “Antibody-Based Inhibition of Pathogenic New World Hemorrhagic Fever Mammarenaviruses by Steric Occlusion of the Human Transferrin Receptor 1 Apical Domain”

**DOI:** 10.1128/jvi.02163-21

**Published:** 2022-02-23

**Authors:** Sol Ferrero, Maria D. Flores, Connor Short, Cecilia A. Vazquez, Lars E. Clark, James Ziegenbein, Samantha Zink, Daniel Fuentes, Cristian Payes, María V. Batto, Michael Collazo, Cybele C. García, Jonathan Abraham, Sandra M. Cordo, Jose A. Rodriguez, Gustavo Helguera

**Affiliations:** a Laboratory of Pharmaceutical Biotechnology, Instituto de Biología y Medicina Experimental (IBYME-CONICET), Buenos Aires, Argentina; b Department of Chemistry and Biochemistry, UCLA-DOE Institute for Genomics and Proteomics, University of California Los Angeles, Los Angeles, California, USA; c Laboratorio de Virología, Departamento de Química Biológica, Facultad de Ciencias Exactas y Naturales, Universidad de Buenos Aires (UBA), IQUIBICEN, Consejo Nacional de Investigaciones Científicas y Técnicas (CONICET)-UBA, Ciudad Universitaria, Buenos Aires, Argentina; d Department of Microbiology, Blavatnik Institute, Harvard Medical School, Boston, Massachusetts, USA; e Department of Biological Chemistry and Department of Chemistry and Biochemistry, University of California Los Angeles, Howard Hughes Medical Institute, UCLA-DOE Institute for Genomics and Proteomics, Los Angeles, California, USA; f Department of Medicine, Division of Infectious Diseases, Brigham and Women’s Hospital, Boston, Massachusetts, USA; g IQUIBICEN, Consejo Nacional de Investigaciones Científicas y Técnicas (CONICET)-UBA, Ciudad Universitaria, Buenos Aires, Argentina

## AUTHOR CORRECTION

Volume 95, no. 17, e01868-20, 2021, https://doi.org/10.1128/JVI.01868-20. Page 15: The following should be added to Acknowledgments after paragraph 2.

This work is based on research conducted at the Northeastern Collaborative Access Team beamlines, which are funded by the National Institute of General Medical Sciences, National Institutes of Health (grant P30 GM124165). The Eiger 16M detector on the 24-ID-E beamline is funded by an NIH-ORIP HEI grant (S10OD021527). This research used resources of the Advanced Photon Source, a U.S. Department of Energy (DOE) Office of Science User Facility operated for the DOE Office of Science by the Argonne National Laboratory under contract no. DE-AC02-06CH11357.

